# Maternal Factors Associated with Levels of Fatty Acids, Specifically n-3 PUFA during Pregnancy: ECLIPSES Study

**DOI:** 10.3390/nu13020317

**Published:** 2021-01-22

**Authors:** Estefania Aparicio, Carla Martín-Grau, Cristina Bedmar, Núria Serrat Orus, Josep Basora, Victoria Arija, The ECLIPSES Study Group

**Affiliations:** 1 Nutrition and Public Health Unit, Research Group on Nutrition and Mental Health (NUTRISAM), Faculty of Medicine and Health Science, Universitat Rovira i Virgili, 43201 Reus, Spain; estefania.aparicio@urv.cat (E.A.); cgmartin.hj23.ics@gencat.cat (C.M.-G.); cristina.bedmar@urv.cat (C.B.); 2 Pere Virgili Institute for Health Research (IISPV), Universitat Rovira i Virgili, 43003 Tarragona, Spain; 3 Clinical Chemistry Laboratory, Catalan Institute of Health (ICS)-Camp de Tarragona-Terres de l’Ebre, Joan XXIII University Hospital in Tarragona, 43005 Tarragona, Spain; nserrat.tarte.ics@gencat.cat; 4 Tarragona-Reus Research Support Unit, Jordi Gol Primary Care Research Institute, 43003 Tarragona, Spain; jbasora.tarte.ics@gencat.cat; 5 CIBERobn (Center for Biomedical Research in Physiopathology of Obesity and Nutrition), Instituto de Salud Carlos III, 28029 Madrid, Spain

**Keywords:** maternal fatty acid status, polyunsaturated fatty acids, omega-3, pregnancy, lifestyle

## Abstract

An optimal fatty acid (FA) profile during pregnancy, especially docosahexaenoic acid (DHA) and eicosapentaenoic acid (EPA), is essential for the health of the mother and child. Our aim was to identify the socioeconomic and maternal lifestyle factors associated with serum FA concentration in pregnant women. A longitudinal study was conducted on 479 pregnant women, who were assessed during the first (T1) and third (T3) trimesters of pregnancy. Data on maternal characteristics, food consumption, and lifestyle were collected. Serum FA concentrations were analysed by a gas chromatography–mass spectrometry combination. The multiple linear regression showed that high educational level and older age were significantly associated with higher EPA and DHA concentrations and lower values of n-6/n-3 and arachidonic acid (AA)/EPA in T1 and/or T3. Regarding diet—fish and seafood consumption increased EPA concentration and reduced n-6/n-3 and AA/EPA values in both trimesters, whereas its consumption increased DHA concentration only in T1. Smoking was associated with lower DHA concentration in T1 and higher values of n-6/n-3 ratio in both trimester. Overweight and obesity were associated with higher values of n-6/n-3 ratio and AA/EPA ratio in T1. A statistically non-significant association was observed with saturated fatty acids (SFA) and monounsaturated fatty acids (MUFA). In conclusion, high educational levels, older age, fish, seafood consumption, and/or non-smoking, are factors that influence better omega-3 polyunsaturated fatty acid (n-3 PUFA) profile in both trimesters of pregnancy. Further research is needed to go in-depth into these findings and their health consequences.

## 1. Introduction

Maternal nutritional status has a crucial role in the outcome of mother and child [1,2,3]. An optimal long-chain n-3 fatty acid status during pregnancy is of particular benefit for cognitive and visual development of the foetus [4,5,6], and is linked to a reduction in the numbers of preterm births and low birth weights, and a reduced risk of preeclampsia and postpartum depression for the mother [1,7,8]. Furthermore, although linoleic acid (LA), omega-6 polyunsaturated fatty acid (n-6 PUFA), is an essential fatty acid, it is suggested that an adequate balance with n-3 is necessary since high levels of n-6 before or during pregnancy may have negative effects on foetal development and may influence the overall health of offspring in later stages of life [9,10].

Nevertheless, the maternal concentration of polyunsaturated fatty acid (PUFA) varies according to different factors. It is known that the maternal diet is one contribution to fatty acid storage [8,11,12] although it could be affected by other factors that have been less studied. Some studies suggest that several factors, such as age, socioeconomic status, education, smoking, and levels of physical activity can all affect fatty acid levels in pregnant women [8,12,13,14,15,16,17], although findings differ. For instance, Hoge et al. [13], in a cohort of 112 pregnant women in Belgium, found nationally that, age, educational level, smoking status, physical activity, and docosahexaenoic acid (DHA) supplement intake, all impacted omega-3 polyunsaturated fatty acid (n-3 PUFA) levels in erythrocytes in the first trimester. Similarity, Gellert et al. [14], in a cohort of 213 pregnant women in Germany, found that smoking impacted negatively on omega-3 index in late pregnancy, although this was not found to be linked with age or region. Markus et al. [8] did not find any association with age in a sample of 118 pregnant women in Norway, but found a link between DHA erythrocytes and fish consumption and educational level. Moreover, Pinto et al. [12] reported from Brazil that age, low income, and weekly fish consumption was a predictor of better n-3 serum concentrations in plasma phospholipids. In addition, a recent systematic review assessed the influence of maternal characteristics on DHA and other polyunsaturated fatty acids during pregnancy [18]. That study identified educational level, maternal age, fish consumption, smoking, alcohol intake, and fatty acid desaturase genotype to be associated with omega-3 maternal status. However, not all studies consider all factors together, and have also been carried out at different moments of the pregnancy. Inconsistent results have been reported with n-6 PUFA and maternal factors [8,12,13,18], and to our knowledge, no study has assessed the link between saturated fatty acids (SFA) and monounsaturated fatty acids (MUFA) in a larger sample size than the present study. Consequently, evidence is scarce about the effect of maternal factors on the serum profile of FA as a whole.

Considering the above factors, a better understanding of the factors associated with an inadequate level of fatty acids throughout the pregnancy period would allow population groups at risk to be targeted in order to recommend improvements to their lifestyle and dietary needs. Thus, the aim of this study was to assess the association between the socioeconomic and maternal lifestyle factors early in pregnancy and the maternal fatty acid profile (saturated, monounsaturated and polyunsaturated fatty acids) from a sample of healthy pregnant women in a European Mediterranean country.

## 2. Materials and Methods

### 2.1. Study Design and Population

A prospective study of a cohort of pregnant women who were followed from the first trimester (T1) (around the 12th gestational week) to the third trimester (T3) (the 36th gestational week). The women were recruited from the ECLIPSES study [19,20], which is a randomized triple-blind clinical trial of different doses of iron supplementation (20 mg/day, 40 mg/day 80 mg/day), registered in ClinicalTrials.gov identification number NCT03196882, and in the European Union (EU) Clinical Trial Register, EUCTR-2012-005480-28. This study was approved by the Clinical Research Ethics Committee of the Jordi Gol Institute for Primary Care Research (IDIAP) and the Pere Virgili Institute for Health Research (IISPV). All participants signed an informed consent. Healthy, pregnant women were recruited during their first prenatal visits at 12 sexual and reproductive health care services (ASSIR) of the Catalan Institute of Health (ICS) in Tarragona, Spain.

Inclusion criteria: healthy woman older than 18 years at ≤12 weeks of gestation, able to understand the local languages (Spanish or Catalan) and the characteristics of the study, and signed the informed consent forms. 

Exclusion criteria: multiple pregnancies, taking iron supplements before week 12 of pregnancy, hypersensitivity to egg protein, previous severe disease (immunosuppression), or any chronic disease that could affect nutritional status (cancer, diabetes, malabsorption, or liver disease). Fatty acid biochemical status was analysed in a total population study of 479 pregnant women.

### 2.2. Data Collection

Data were collected during T1 and T3 by midwives and nutritionists. The medical history and socioeconomic data obtained were maternal age, ethnicity, education level (primary, secondary, and university studies), estimated date of delivery, planned pregnancy, clinical and obstetric history, and taking multivitamins and micronutrient supplements. Socioeconomic level was classified as low, middle, or high, according to the Catalan classification of occupations [21]. Lifestyle habits were recorded, including smoking habit and alcohol intake, and physical activity levels were assessed by means of the short version of the International Physical Activity Questionnaire (IPAQ-S) [22]. Women were classified as sedentary (including sedentary and irregularly active women) or active (including active and very active women), according to the modified Craig algorithm [22]. In addition, anthropometric measurements obtained were height (cm) and weight (kg), and body mass index (BMI) was calculated at week 12. Thus, women were classified as normal weight (BMI < 25 kg/m^2^) and overweight (BMI ≥ 25 kg/m^2^), according to the World Health Organization (WHO) criteria [23].

Diet was assessed using a self-administered food frequency questionnaire (FFQ) validated for our population [24]. The questionnaire contains 45 food items and reports on the usual food consumption per week or per month. The food items were classified as fish and seafood, red and processed meat, lean meat, fruit (fresh fruit, preserved fruit), vegetables (salads and vegetables), dairy products, legumes, cereals (breakfast cereals, bread, pasta, and rice), bakery (biscuits, pastries), nuts, sweets (sugar and chocolates), sweetened beverages, alcoholic drinks. Fat consumption, especially oil olive, took into account the amount of oil used for cooking or salad dressings. Food consumption was calculated per gram per day. The consumption of those foods relevant due to their fatty acid content (such as fish and seafood, nuts, red and processed meat, lean meat, eggs, bakery and oil) were included in the analysis. Diet quality was assessed using the Relative Mediterranean Diet Score [25,26], ranging from 0 points (low quality diet) to 18 points (high quality diet). Further information can be found in our previous paper [27]. Women were then classified into two categories: low-medium diet quality (score from 0 to 10) and high diet quality (score from 11 to 18).

### 2.3. Sample Collection and Processing of Biochemical Samples of Fatty Acids, and Extraction, Transfer and Storage of Biological Samples

Venous blood samples were collected at weeks 12 and 36 of pregnancy, after fasting, into 7.5 mL tubes without an anticoagulant, and remained without mixing for 30 min at room temperature so as to allow coagulation. The serum was separated by centrifugation, distributed into aliquots of 500 μL and stored at −80 °C until assays. Samples were stored in the BioBank and thawed at the end of the clinical study and processed simultaneously to minimize inter-batch variation [19].

Medium- and long-chain fatty acids (saturated-, mono- and polyunsaturated) were measured by gas chromatography–mass spectrometry (GC-MS) combination using the 7890A GC coupled to QqQ 7000 Series^®^ (Agilent Technologies Inc., Santa Clara, CA, USA) after their derivation to methyl ester (FAMEs) due to their higher volatility [28]. Briefly, a 50 μL plasma sample was mixed with internal standard (IS) solution (Myristic d-27 acid, Merck KGaA, Darmstadt, Germany), chloroform and methanolic hydrochloric acid and incubated at 80 °C for 2 h. Obtained FAMEs were extracted by a liquid–liquid extraction using hexane and were then injected into the GC-MS system. Chromatographic analysis was based on David et al. [28] to determine the 36 FAMEs included in the Food Industry FAME Mix (Restek Corporation, Pennsylvania, USA). FAMEs were separated into a high-polarity column (100 m × 250 μm × 0.25 μm) (HP-88 column, Agilent Technologies Inc., Santa Clara, CA, USA) using a temperature program of between 140 °C and 240 °C at 1 mL/min using helium as the carrier gas. Ionisation was carried out by electronic impart (70 eV) and mass analyser was operated in Selected Ion Monitoring mode (SIM). The CG-MS system was controlled by the Agilent MassHunter Workstation.

A total of 36 fatty acids were analysed, but only a selection of them is presented, such as the sum of the total saturated (total SFA = C:12 + C:14 + C:16 + C18:0), total monounsaturated (total MUFA = C16:1n-7 + C18:1n-9), total n-6 polyunsaturated (total n-6 PUFA = C18:2n-6 + C20:3n-6 + C20:4n-6) and total n-3 polyunsaturated (total n-3 PUFA = C20:5n-3 + C22:6n-3) fatty acids; further, the ratio of the n-6 to n-3 fatty acids (total n-6 PUFA/total n-3 PUFA) are also presented.

### 2.4. Statistical Analysis

The results were expressed as mean ± standard deviation (SD) or percentage according to the variable. In this study, z-score analysis was used to detect outlier value in this population data [29]. An absolute value of ±3.29 is the standard value used to identify outliers when sample size is >100. In other words, any z-score above or below ±3.29 is considered as an outlier case [30]. Means between groups were compared using Student’s t-test or one-way analysis of variance (ANOVA) adjusted by Bonferroni. Multiple linear regressions were performed in order to identify the association of maternal factors, assessed in T1 and T3, with fatty acid concentration in T1 and T3, respectively. All multiple linear regressions models were performed using the ENTER method and were adjusted for maternal factors, such as educational level, maternal age, BMI, smoking status, alcohol consumption, physical activity, diet quality, food consumption (fish and seafood, nuts, red and processed meat, lean meat, eggs, bakery, and oil). In addition, the models were adjusted for intervention group (iron supplementation of 40, 20, or 80 mg/day). Data were processed using the statistical software package SPSS version 25.0 for Windows (SPSS, Chicago, IL, USA). A *p*-value < 0.05 was considered statistically significant.

## 3. Results

### 3.1. Participants’ Characteristics

General characteristics of the pregnant women are given in Table 1. Most women had a medium educational level (38.3%) and were employed (87.1%). The mean maternal age was 30.6 ± 5.01 years. The participants reported that 15.3% smoked at the beginning of their pregnancy and 92% were rated as sedentary by the IPAQ-S questionnaire. The mean score of diet quality was 9.7 (±2.6) points. In addition, 67.9% of women reported taking multivitamin and mineral supplementation, of which only one contained DHA or fish oil, and was only consumed by one woman.

### 3.2. Influence of Maternal Factors on Fatty Acid Serum Profiles

Regarding maternal factors shown in Table 2, some of them were related to SFA, n-6 PUFA and MUFA. Overweight or obese women showed a significantly higher concentration of total FAs, specifically SFA and arachidonic acid (AA), total n-6 PUFA and n-6/n-3 ratio in T1, and only SFA in T3. Women with a higher educational level had a significantly greater concentration of total n-3 PUFA (eicosapentaenoic acid (EPA) + DHA) in serum and significantly lower values of n-6/n-3 ratio and AA/EPA ratio compared to women with low and medium educational level in both trimesters of pregnancy. Moreover, older women (34.4 ± 2.7 years old) had a significantly higher concentration of total n-3 PUFA in serum and lower values of n-6/n-3 ratio and AA/EPA ratio in both trimesters. However, only during T3, older women had a significantly greater concentration of total MUFA compared to younger women. Depending on smoking status, the following differences were observed: women who smoked had a significantly lower concentration of total n-3 PUFA, EPA, and DHA in T1; smokers had a significantly higher concentration of total SFA, total n-6 LA, AA, and total FAs in T3 compared to non-smokers. Moreover, smokers had a higher value of n-6/n-3 ratio in both trimesters. However, there was no observed association with maternal alcohol consumption.

Multiple linear regression models of the influence of maternal determinants on EPA, and DHA values and total n-3 FA are shown in Table 3 and n-6/n-3 ratio and AA/EPA ratio are described in Table 4. It can be observed that women of a high educational level and an age above 30 years had significantly higher levels of EPA in both trimesters of pregnancy. Likewise, EPA values were higher by fish and seafood consumption in both trimesters (*p* < 0.05). Moreover, high educational level, older age, and fish and seafood consumption is significantly associated with higher values of DHA in T1. Alternatively, smoking reduced the concentration of DHA to around 40 µmol/L in T1 (*p* < 0.05). In T3, ages above 25 or 30 predicted higher values of DHA, although the model is not significant. In the same way, total n-3 is significantly higher in T1 at higher levels of education, age above 30 years and fish and seafood consumption, whereas it is lower for smokers (Table 3). In T3, only age between 25 and 30 years and older than 30 years is associated with higher values of total n-3 (Table 3). In addition, iron supplementation of 80 mg/day is associated with higher values of EPA, DHA, total n-3 in T1.

Regarding n-6/n-3 ratio and AA/EPA, high educational level in T1 and T3 reduces the n-6/n-3 ratio. On the other hand, medium and high educational level and age older than 25 or 30 decreased AA/EPA ratio in both trimesters (except for age group between 25 and 30 in T3). Moreover, fish and seafood consumption is associated with significantly lower values of n-6/n-3 ratio and AA/EPA in both trimesters. As expected, smoking increased the n-6/n-3 ration by around 2 and 4 points in T1 and T3, respectively. Moreover, overweight or obesity increased the n-6/n-3 ratio and AA/EPA ratio, while iron supplementation of 80 mg/day reduced AA/EPA ratio in T1 (Table 4). In addition, there was no statistically significant association between maternal factors and AA concentration (data not shown).

## 4. Discussion

In this prospective study of Mediterranean pregnant women, we identified the main maternal factors that affect the n-3 PUFA and their balance with n-6 PUFA. Specifically, women of 25 or 30 years old and/or with medium or high educational level showed better levels of fatty acids, with a greater n-3 fatty acids serum concentration and lower values of n-6/n-3 and AA/EPA ratio in both the first (T1) and third (T3) trimesters. Moreover, higher fish and seafood consumption is favourably linked with higher EPA and DHA concentrations and with a lower n-6/n-3 ratio and AA/EPA in both trimesters, except for DHA, which was not linked in T3. However, smoking reduced DHA concentration and increased n-6/n-3 ratio T1, but only n-6/n-3 in T3. Practically none of the maternal factors assessed were related to SFA, apart from overweight and obesity status, or MUFA, apart from the older age of the mother in T3.

To our knowledge, there are few studies that have assessed maternal and lifestyle factors associated with serum fatty acid concentrations in a pregnant population, DHA and EPA being of great importance for mother and child health [31]. Moreover, an adequate balance between n-3 and n-6 is a critical factor for health because the n-6 PUFA compete with the n-3 PUFA by the metabolism of desaturation enzymes and, consequently, influences the kind of eicosanoid generated [32]. It is relevant to evaluate the association of FA serum concentrations and the maternal factors related throughout the whole pregnancy period. Specifically, diet is one of the most studied factors related to FA status. Nowadays, the occidental diet tends not to sufficiently provide n-3 PUFA, and the n-6 PUFA intake tends to be higher than recommendations [33,34]. The main source of n-3 PUFA are fish and seafood, which could be crucial contributors to an adequate level of n-3 PUFA and the n-6/n-3 and AA/EPA ratios during pregnancy, as has been reported previously [8,11,12,18]. As expected, our results showed that fish and seafood consumption positively predicted greater serum concentrations of EPA and DHA and low values of n-6/n-3 ratio and AA/EPA ratio in T1. Within estimations based on our model, it was pointed out that for each fish serving (150 g of weight approximately), the concentration of EPA and DHA increases by 22.5 and 51 µmol/L respectively, improving their concentrations considerably. However, these associations were only maintained for EPA and n-6/n-3 and AA/EPA ratios during T3, but not for DHA concentrations in agreement with Bonham et al. [35]. This might be due to the fact that DHA could be regulated by different biosynthesis or mobilisation mechanism [34]. For instance, hormonal changes that occur during pregnancy, such as the increase in oestrogen that supports conversion of alpha-linolenic acid (ALA) to DHA [36,37,38]. Another reason might be that in T3 there is a high mobilisation of DHA from the maternal fat store to the foetus in order to stimulate high brain maturation. In addition, bakery food, which is usually manufactured with vegetable oils rich in n-6, can predict high AA/EPA ratios. In view of these findings, an optimal n-3 PUFA concentration and n-6/n-3 and AA/EPA ratios at the beginning of the pregnancy are needed to assure an adequate concentration and store throughout the pregnancy and even the lactation period [8]. Therefore, to acquire healthy eating habits, including fish and seafood consumption, it is essential to achieve an optimal EPA and DHA concentration at the beginning and during pregnancy, which could contribute to improving child neurodevelopment and protection from autism-spectrum traits [39,40,41]. In particular, higher maternal n-6/n-3 ratios and higher maternal concentrations of total n-6 PUFA are among potential environmental risk factors that are associated to autism spectrum disorder [39,42] and immune system disease [43]. The placental fat transport is driven by a concentration gradient as the foetus has substantially lower fat concentrations, indicating a preferential transfer of n-6 PUFA [42]. The consequence is an imbalance between n-3 and n-6 PUFA levels that may contribute to offspring diseases. We found that age and educational level are also the common contributors to fatty acid concentrations in maternal serum throughout pregnancy. Our results show that those women over 25 years of age, or with medium or high educational level in particular, showed high values of EPA and DHA and low values of n-6/n-3 and AA/EPA ratio. Some authors have not reported a significant association between age or educational level and DHA or total n-3 PUFA [8,14], although similar findings to ours regarding age have been reported in different countries [12,13,15,17]. Likewise, Pinto et al. [12] found that low income correlated negatively with the n-6/-n-3 ratio, and other studies have reported a positive correlation between educational level and levels of DHA, total n-3 PUFA and a negative correlation with AA/EPA ratio [13,16]. Although some research has shown that older women with a higher educational level tend to consume more n-3 or fish and seafood during pregnancy [44,45], and/or showed high adherence to diet quality [27,46,47], in our study only observed significant differences on fish and seafood consumption by age group (age group: <25 years old: 36.3 ± 21.6; 25–30years old: 46.1 ± 31.6; >30 years old: 47.2 ± 30.5; *p* = 0.049). In addition, women with medium or high educational levels might have greater nutritional knowledge [48,49]. Lower educational levels and younger ages might be linked to lower incomes and may have less access to food sources of n-3 PUFA (fish and seafood), which tend to be expensive [12,50,51]. In agreement with our results, Nordgren et al. [51] indicates that socioeconomically disadvantaged populations (low educational level and low income) are particularly at risk for even lower levels of omega-3 intake. Regarding n-6 PUFA, two studies also report no link with age [8] or educational level [13] in agreement with our results. In contrast, two studies observed a positive link with age [12,16], but others showed a negative correlation with age [13,16] and/or education level [8].

Nutritional status could be a potential factor, although the literature shows controversial results [11,12,13,14,15]. Lesch et al. [11] found overweight women showed higher concentrations of AA and n–3 PUFA. However, our results show that overweight or obesity status is associated to higher concentrations of SFA, AA andn-3/n-6 and AA/EPA ratio. It can be hypothesized that, in our sample, the higher FA level might due to greater food consumption, especially of those foods rich in n-6 FA, which could interfere in the conversion of EPA. Moreover, excessive maternal adiposity could alter the placental transfer of FA [52].

Another of our principal relevant findings is that pregnant women who smoke showed low levels of DHA, total n-3 and high n-6/n-3 ratio only in T1. In particular, smoking predicted a reduction of around 40 µmol/L of DHA. These results confirm recent findings in the omega-3 index in erythrocyte or in total n-3 in serum fatty acid [12,13,14,15,17,53]. This influence is possibly caused by the adverse effect of smoking on the conversion process of ALA to DHA [54,55]. This link with DHA and n-3 was not observed in T3, probably by the mechanism of transferring DHA maternal stores to foetus. However, it was shown that smoking increased the n-6/n-3 ratio, which suggests that smoking could affect the fatty acid profile some another way since it might alter the conversion rate of n-6 to eicosanoids, such as prostaglandin E2 [55,56]. This shows an anti-inflammatory effect and has a crucial role in the regulation of delivery [57]. In fact, smoking during pregnancy has been shown as a risk factor for premature delivery [58]; therefore, advising a mother to quit smoking in pregnancy could improve the FA profile throughout gestation and prevent negative outcomes for mother and child.

Regarding another unhealthy lifestyle habit, some studies have found that maternal alcohol consumption has been associated with worse n-6 and n-3 FA concentrations [12,15,16,18,53] since it could impair lipid metabolism [59]. However, our results did not find any significant association, probably due to the fact that the amount of alcohol consumption in our sample is low (3.01 g/day ± 15.2) in comparison to women who consume alcohol frequently [16] or moderately-heavily [53].

It should be noted that iron supplementation of 80 mg was associated to higher concentration of EPA, DHA total n-3 and a lower AA/EPA ratio. There appears to be a physiological relationship between the metabolism and utilization of iron and that of fatty acids, although it is not yet clear [60].

Our findings provide crucial information for knowing and identifying maternal factors related to serum concentrations of FA and developing an intervention program focused on modifiable factors, such as dietary and smoking habits, and targeting high-risk groups. Our study has several strengths. It is longitudinal, which enabled us to study the concentrations of fatty acid and the maternal factors throughout the pregnancy period. Secondly, all procedures in data collection and sample analyses were intensively monitored. Moreover, we included in our analysis different types of FA. Indeed, this is the first study that has analysed SFA and MUFA with several maternal factors. Another strength is the greater sample size in comparison with other studies [12,13,14].

However, our study also has several limitations. Firstly, due to the lack of references or range values, we were not able to apply a cut-off value to describe the intensity of how maternal factors affect the fatty acid profile and which potential consequences of lower n-3 status can appear in the mother and child. Further studies regarding the whole FA profile are encouraged. On the other hand, although we had data on fatty acid concentrations in plasma rather than red blood cells, we consider that our results are not affected because it has been shown that fatty acid concentrations in plasma and red blood cells are highly correlated [61,62,63] and studies that use both biochemical parameters obtained similar results [15]. Finally, we were unable to evaluate other environmental and genetic factors that might impact on serum fatty acid concentrations during pregnancy. For instance, gene coding for proteins involved in uptake, metabolism, transport and restructuration, especially, the fatty acid desaturase (FADS) gene cluster, which is related to desaturation steps in the n-3 and n-6 fatty acids might also contribute [64].

## 5. Conclusions

In conclusion, educational level or age are common factors that affected EPA, DHA, and the n-6/n-3 and AA/EPA in both trimesters. Overweight and obesity predicted higher values of n-6/n-3 ratio and AA/EPA ratio in the first trimester. Lifestyle habits, such as fish and seafood consumption, increased EPA and AA/EPA ratio in both trimesters, whereas DHA was modified by fish and seafood consumption in the first trimester. Smoking showed an impairment in the fatty acid profile: a decrease in DHA in the first trimester and an increase of n-6/n-3 in both trimesters. However, no relevant association was found with SFA and MUFA. Therefore, nutritional intervention and advice to stop smoking geared to pregnant women, especially those who are younger than 25 years-old, with low educational levels, or are overweight or obese, could improve the FA serum profiles, especially n-3 PUFA, as it may benefit their health and the health of their newborns. Further research is needed to go in-depth into these findings and their health consequences.

## Figures and Tables

**Table 1:** Sociodemographic and lifestyle of pregnant women at baseline and fatty acid biochemical profile at first trimester.

General Characteristics	Mean (SD)
Maternal age (years) ^a^ (*n* = 461)	30.6 ± 5.01
Age group (%)	
<25 years old	15.8
25–30 years old	31.2
>30 years old	52.9
BMI (kg/m^2^) (%) (*n* = 455)	
<25	62.2
25–30	25.3
≥30	12.5
Maternal educational level (%) (*n* = 478)	
Low (primary or less)	30.1
Medium (secondary)	38.3
High (university or more)	31.6
Occupation (%) (*n* = 459)	
Student	2.4
Employed	87.1
Unemployed	10.5
Smoking status (%) *(n* = 478)	
Smoker	15.3
Non-Smoker or Ex-Smoker	84.7
Maternal alcohol consumption (%) (*n* = 445)	14
Physical Activity (%) (*n* = 450)	
Active	8
Sedentary	92
Diet Quality (score)	9.7 (2.6)
Food groups	
Fish and seafood consumption (g/day)	45.2 (30.5)
Lean meat (g/day)	39.4 (24.7)
Red and processed meat (g/day)	57.5 (32.3)
Eggs (g/day)	17.4 (11.3)
Bakery (g/day)	33.7 (28.2)
Nuts (g/day)	3.1 (3.8)
Oil (g/day)	63.8 (15.1)

^a^ Values are expressed as mean and standard deviation (SD) or %. Abbreviation: BMI, body mass index.

**Table 2:** Fatty acids serum profile during the first (T1) and third (T3) trimester of pregnancy according to maternal factors.

Fatty Acids (µmol/L)			Physical Activity			BMI			
		All Woman (*n* = 479)	Active ^a^ (*n* = 35)	Sedentary ^b^ (*n* = 404)	*p*-Value ^a,b^	Normal Weight (<25 kg/m^2^) ^c^ (*n* = 283)	Overweight and Obesity (>25kg/m^2^) ^d^ (*n* = 172)		*p*-Value ^c,d^
		Mean (SD)	Mean (SD)	Mean (SD)		Mean (SD)	Mean (SD)		
Total SFA	1 T	3765.31 (1614.17)	3793.8 (1829.6)	3740.1 (1530.1)	0.849	3647.5 (1480.4)	4131.3 (1897.4)		0.006
	3 T	9625.65 (4574.33)	9590.3 (4656.8)	9641.2 (4512.4)	0.950	9299.9 (4527.0)	10,380.9 (4637.3)		0.017
Total MUFA	1 T	1634.54 (500.21)	1652.7 (678.1)	1631.3 (467.3)	0.806	1607.8 (485.5)	1734.6(549.3)		0.013
	3 T	3116.39 (1330.86)	3114.7 (1550.3)	3140.5 (1312.9)	0.913	3038.8 (1228.4)	3275.3 (1462.9)		0.084
n-6 PUFA									
LA (C18:2n-6)	1 T	3355.67 (1230.19)	3467.6 (1327.0)	3337.1 (1199.4)	0.546	3309.9 (1210.9)	3537.9 (1280.5)		0.064
	3 T	6321.14 (2786.02)	6717.1 (3482.0)	6239.9 (2629.7)	0.428	6271.3 (2869.6)	6475.0 (2532.5)		0.451
AA (C20:4n-6)	1 T	830.82 ± 276.15	780.4 (262.4)	838.1 (281.7)	0.250	817.3 (262.1)	889.8 (296.7)		0.008
	3 T	722.63 ± 220.05	734.6 (272.8)	723.6 (216.3)	0.815	709.1 (206.1)	750.6 (235.2)		0.062
Total n-6 PUFA	1 T	4433.77 ± 1469.64	4495.1 (1648.1)	4410.8 (1424.7)	0.744	4358.2 (1445.1)	46.70.3 (1485.9)		0.032
	3 T	7278.06 ± 2919.04	7431.4 (3411.6)	7220.8 (2784.3)	0.725	7187.0 (2960.1)	7513.1 (2711.3)		0.248
n-3 PUFA									
EPA (C20:5n-3)	1 T	35.03 (23.95)	28.7 (16.0)	36.8 (25.9)	0.010	36.1 (25.5)	35.7 (24.2)		0.879
	3 T	23.88 (16.93)	20.5 (16.5)	24.2 (16.9)	0.211	23.1 (16.1)	24.1 (17.2)		0.513
DHA (C22:6n-3)	1 T	240.28 (73.47)	221.3 (72.3)	241.6 (73.5)	0.128	239.7 (72.8)	248.1 (78.9)		0.253
	3 T	236.60 (71.77)	229.6 (80.6)	237.6 (71.0)	0.525	233.9 (69.0)	237.2 (72.2)		0.631
Total n-3 PUFA	1 T	276.98 (94.07)	258.0 (97.5)	279.1 (94.2)	0.212	277.0 (93.7)	287.4 (102.8)		0.277
	3 T	260.52 (84.33)	253.0 (91.6)	261.9 (84.5)	0.549	257.2 (80.6)	261.8 (86.0)		0.574
Total FAs	1 T	10,073.15 (3349.07)	10,462.6 (4446.0)	10,038.2 (3175.5)	0.589	9781.9 (3045.6)	10,888.1 (3848.4)		0.002
	3 T	20,480.82 (8335.23)	20,792.6 (9549.0)	20,406.6 (8054.6)	0.792	19,856.2 (8006.0)	21,814.7 (8638.8)		0.017
n-6/n-3 ratio	1 T	16.80 (5.19)	17.9 (4.6)	16.7 (5.2)	0.182	16.5 (5.0)	17.7 (5.5)		0.016
	3 T	29.29 (11.52)	30.9 (11.9)	29.2 (11.4)	0.408	26.3 (11.6)	30.4 (11.6)		0.360
AA/EPA ratio	1 T	31.77 (19.08)	35.6 (21.1)	31.2 (18.7)	0.184	31.0 (19.3)	33.4 (18.8)		0.197
	3 T	44.09 (30.61)	52.1 (33.4)	44.3 (31.6)	0.158	45.2 (32.4)	46.9 (31.8)		0.581
**Fatty Acids**		**Maternal Smoking Status**				**Maternal Alcohol Consumption**			
		**Smoker ^e^** **(*n* = 70)**	**Non-Smoker or Ex-Smoker ^f^** **(*n* = 390)**		***p*-Value ^e,f^**	**No ^g^** **(*n* = 380)**	**Yes ^h^** **(*n* = 65)**		***p*-Value ^g,h^**
		**Mean (SD)**	**Mean (SD)**			**Mean (SD)**	**Mean (SD)**		
Total SFA	1 T	3896.7 (1840.4)	3795.3 (1616.2)		0.635	3846.7 (1721.4)	3552.7 (1248.4)		0.192
	3 T	10,992.9 (5035.4)	9421.6(4438.9)		0.008	9756.5 (4684.2)	9735.7 (4291.2)		0.974
Total MUFA	1 T	1592.1 (483.6)	4737.4 (1514.7)		0.309	1661.0 (526.9)	1596.0 (455.2)		0.355
	3 T	3369.1 (1239.0)	3086.8(1349.4)		0.102	3142.1 (1345.6)	3139.6 (1251.1)		0.989
n-6 PUFA									
LA (C18:2n-6)	1 T	3378.5 (1289.9)	3374.4 (1226.1)		0.980	3407.8 (1251.6)	3282.8 (1191.9)		0.459
	3 T	7108.4 (2856.9)	6185.8 (2709.2)		0.010	6368.9 (2777.7)	6540.3 (2689.0)		0.652
AA (C20:4n-6)	1 T	812.5 (278.5)	842.0(278.3)		0.411	848.6 (278.0)	811.8 (240.3)		0.321
	3 T	781.7 (251.8)	713.9(212.3)		0.017	721.4 (218.1)	758.4 (223.9)		0.216
Total n-6 PUFA	1 T	4430.2 (1518.5)	4450.7(1461.5)		0.915	4495.4 (1489.0)	4320.3 (1374.0)		0.356
	3 T	8026.8 (2904.0)	7155.0 (2856.1)		0.020	7320.8 (2904.8)	7552.7 (2827.1)		0.560
n-3 PUFA									
EPA (C20:5n-3)	1 T	29.9 (20.5)	37.1(25.6)		0.010	35.9 (24.8)	38.3 (27.4)		0.477
	3 T	21.8 (16.3)	24.1(16.8)		0.278	23.5 (16.3)	23.9 (18.2)		0.845
DHA (C22:6n-3)	1 T	212.0 (67.3)	247.4 (74.2)		<0.001	244.0 (74.5)	235.7 (73.9)		0.415
	3 T	225.3 (69.3)	237.7(72.1)		0.181	233.5 (68.9)	243.8 (82.8)		0.352
Total n-3 PUFA	1 T	241.9 (80.5)	287.0(97.1)		<0.001	282.1 (96.6)	274.1 (94.2)		0.538
	3 T	247.1 (81.1)	262.3(85.4)		0.163	257.0 (81.0)	268.8 (98.0)		0.368
Total FAs	1 T	9957.3 (3289.8)	10,183.4(3417.6)		0.611	10,235.9 (3489.2)	99,743.1 (2884.8)		0.287
	3 T	22,811.9 (8668.9)	20,111.4(8147.6)		0.012	20,663.8 (8442.9)	20,952.8 (7776.3)		0.800
n-6/n-3 ratio	1 T	19.5 (5.1)	16.4(5.1)		<0.001	16.9 (5.1)	16.6 (5.6)		0.650
	3 T	34.9 (11.7)	28.6 (11.2)		<0.001	29.9 (11.6)	29.4 (11.2)		0.725
AA/EPA ratio	1 T	36.1 (17.6)	31.0(19.3)		0.039	31.9 (19.1)	30.4 (19.3)		0.565
	3 T	50.0 (31.2)	44.6(32.2)		0.195	45.9 (31.9)	45.2 (33.1)		0.882
**Fatty Acids**		**Educational Level**				**Age Group (Years)**			
		**Low ^i^** **(*n* = 137)**	**Medium ^j^** **(*n* = 175)**	**High ^k^** **(*n* = 147)**	***p*-Value ^i–k^**	**<25 ^l^** **(*n* = 55)**	**25-30 ^m^** **(*n* = 153)**	**>30 ^n^** **(*n* = 233)**	***p*-Value ^l–n^**
		**Mean (SD)**	**Mean (SD)**	**Mean (SD)**		**Mean (SD)**	**Mean (SD)**	**Mean (SD)**	
Total SFA	1 T	3874.1 (1749.7)	3855.9 (1732.5)	3698.3 (1450.3)	0.605	3563.0 (1597.9)	3784.0 (1741.0)	3920.5 (1625.2)	0.342
	3 T	10,292.9 (5143.4)	9364.9 (4260.0)	9400.1 (4277.2)	0.142	8759.2 (4260.8)	9446.6 (4490.7)	10,091.9 (4704.0)	0.104
Total MUFA	1 T	1620.1 (504.1)	1667.6 (539.5)	1655.8 (479.1)	0.707	1542.2 (484.3)	1620.8 (523.8)	1703.5 (508.4)	0.070
	3 T	2971.5 (1196.4)	3115.0 (1369.9)	3308.5 (1410.3)	0.102	2612.1 (1049.3)	3020.6 (1259.4)	3323.5 (1386.8)	0.001
n-6 PUFA									
LA (C18:2n-6)	1 T	3508.0 (1285.4)	3363.6 (1200.5)	3263.9 (1222.1)	0.249	3138.3 (1013.0)	3339.3 (1182.5)	3496.1 (1319.6)	0.132
	3 T	6637.6 (2698.6)	6439.7 (2871.8)	5886.9 (2604.5)	0.055	6334.4 (2834.4)	6372.2 (2730.3)	6338.7 (2740.4)	0.992
AA (C20:4n-6)	1 T	832.3 (252.5)	841.2 (306.5)	837.7 (266.4)	0.961	782.9 (271.7)	844.0 (261.7)	858.8 (287.9)	0.198
	3 T	748.9 (221.8)	714.4 (217.9)	712.9 (220.1)	0.287	683.1 (185.1)	730.8 (212.5)	733.1 (231.4)	0.287
Total n-6 PUFA	1 T	4603.5 (1501.6)	4418.9 (1453.0)	4336.4 (1453.6)	0.397	4122.7 (1251.0)	4439.0 (1428.2)	4589.3 (1539.3)	0.108
	3 T	7526.6 (2662.0)	7405.5 (3021.7)	6913.7 (2876.7)	0.156	7246.7 (2923.9)	7380.0 (2894.9)	7285.0 (2845.2)	0.935
n-3 PUFA									
EPA (C20:5n-3)	1 T	29.0 (19.4)	36.1 (25.3)	42.5 (27.8)	<0.0001	25.7 (22.7)	32.6 (24.2)	40.6 (25.1)	<0.0001
	3 T	19.6 (15.6)	23.4 (15.5)	28.1 (18.2)	<0.0001	14.8 (10.8)	23.0 (16.2)	25.9 (17.3)	<0.0001
DHA (C22:6n-3)	1 T	228.0 (70.0)	238.9 (71.8)	258.6 (78.2)	0.002	207.7 (57.7)	240.8 (79.5)	252.3 (73.4)	<0.0001
	3 T	221.4 (66.6)	238.6 (70.8)	246.5 (75.9)	0.009	199.7 (60.4)	232.1 (66.2)	245.6 (72.2)	<0.0001
Total n-3 PUFA	1 T	259.0 (87.2)	276.2 (90.8)	304.3 (105.1)	<0.0001	233.4 (73.2)	273.4 (98.3)	296.7 (97.5)	<0.0001
	3 T	241.0 (78.6)	262.5 (83.4)	275.5 (89.4)	0.002	215.1 (68.3)	256.7 (80.6)	271.0 (83.8)	<0.0001
Total FAs	1 T	10,307.0 (3389.4)	10,208.3 (3635.0)	9930.1 (3106.4)	0.623	9424.7 (3016.7)	9959.2 (3312.9)	10,538.9 (3524.7)	0.057
	3 T	20,891.2 (7860.5)	20,493.4 (8708.0)	20,207.8 (8177.2)	0.783	18,711.7 (6936.9)	20,099.0 (7990.0)	21,362.9 (8701.4)	0.066
n-6/n-3 ratio	1 T	18.7 (5.3)	17.2 (5.3)	14.7 (4.3)	<0.0001	18.4 (5.1)	17.6 (5.5)	16.2 (5.0)	0.005
	3 T	33.1 (11.4)	29.9 (12.4)	25.7 (9.2)	<0.0001	35.6 (12.7)	30.1 (11.6)	28.1 (10.9)	<0.0001
AA/EPA ratio	1 T	38.0 (20.0)	31.9 (18.8)	26.1 (16.9)	<0.0001	42.4 (24.1)	35.1 (19.6)	27.7 (16.4)	<0.0001
	3 T	58.8 (38.5)	43.5 (30.3)	35.2 (21.7)	<0.0001	64.5 (38.2)	47.7 (34.4)	40.7 (27.7)	<0.0001

Values are expressed as mean and standard deviation (SD). Abbreviation: BMI, body mass index; SFA, saturated fatty acids; MUFA, monounsaturated fatty acids; n-6 PUFA, omega-6 polyunsaturated fatty acid; LA, Linoleic acid; AA, Arachidonic acid; n-3 PUFA, omega-3 polyunsaturated fatty acid; EPA, Eicosapentaenoic acid; DHA, Docosahexaenoic acid; Total FAs, fatty acids = total SFA + total MUFA + total n-6 PUFA + total n-3 PUFA; n-6/n-3 ratio = total n-6 PUFA/ total n-3 PUFA. ^a^ Active; ^b^ Sedentary; *p*-value ^a,b^, *p*-value between active and sedentary women. ^c^ Normal weight; ^d^ Overweight and obesity; *p*-value ^c,d^, p-value between normal weight (<25 kg/m^2^) and overweight and obesity (≥25 kg/m^2^). ^e^ Smoker; ^f^ Non-smoker; *p*-value ^e,f^, *p*-value between smoker or non-smoker women. ^g^ No-alcohol consumption; ^h^ Yes-alcohol consumption; *p*-value ^g,h^, *p*-value between No-alcohol consumption and alcohol consumption. ^i^ Low; ^j^ Medium; ^k^ High; *p*-value ^i–k^, *p*-value between low, medium and high educational level. ^l^ Age less than 25 years-old; ^m^ Age between 25 and 30 years-old; ^n^ Age more than 30 years-old; *p*-value ^l–n^, *p*-value between age groups. Student’s *t*-test was used for Physical activity, BMI, maternal smoking status and maternal alcohol consumption. One-way analysis of variance (ANOVA) was used for educational level and age group.

**Table 3:** Multiple linear regression of potential factors related to maternal serum polyunsaturated fatty acid composition in the first (T1) and third (3T) trimesters of pregnancy.

	Fatty Acids Serum Profile First Trimester			Fatty Acids Serum Profile Third Trimester		
	B	SE	*p*	B	SE	*p*
Eicosapentaenoic acid (EPA)						
Constant	25.13	7.86	0.002	16.46	6.84	0.017
Educational level						
Low	Ref	-	-	Ref	-	-
Medium	7.65	3.32	0.022	0.49	2.62	0.851
High	9.69	3.53	0.006	6.87	2.83	0.016
Age group						
<25 years	Ref	-	-	Ref	-	-
25–30 years	3.75	4.38	0.392	6.79	3.24	0.038
≥30 years	9.93	4.22	0.019	8.80	3.11	0.005
BMI (0: < 25 kg/m^2^, 1: ≥ 25 kg/m^2^)	−1.21	2.80	0.665	0.82	2.60	0.751
Physical activity (0: sedentary, 1: active)	−9.58	4.51	0.035	−2.02	2.51	0.421
Smoking (0: non-smoke, 1: smoker)	−6.61	3.72	0.076	2.85	2.70	0.292
Maternal alcohol consumption (0: no, 1: yes)	−0.86	4.67	0.854	0.61	3.79	0.871
Fish and seafood consumption (g/day)	0.15	0.04	0.001	0.14	0.40	<0.001
Bakery consumption (g/day)	−0.1	0.04	0.037	−0.05	0.041	0.204
Intervention Group (iron supplementation dosage)						
40 mg/day	Ref	-	-	Ref	-	-
20 mg/day	1.70	3.72	0.648	−2.10	2.67	0.432
80 mg/day	7.97	3.07	0.010	−1.78	2.47	0.473
	R^2^_CX100_ = 10.3	F_18,328_ = 3.21	*p* < 0.001	R^2^_CX100_ = 8.8	F_18,234_ = 2.25	*p* = 0.002
Docosahexaenoic acid (DHA)						
Constant	212.64	22.29	<0.001	241.16	30.48	<0.001
Educational level						
Low	Ref	-	-	Ref	-	-
Medium	10.29	9.50	0.280	−2.09	11.06	0.850
High	22.45	10.01	0.026	11.54	12.04	0.339
Age group						
<25 years	Ref	-	-	Ref	-	-
25–30 years	19.46	12.53	0.121	29.46	13.73	0.033
>30 years	28.01	12.07	0.021	41.26	13.23	0.002
BMI (0: < 25 kg/m^2^, 1: ≥ 25 kg/m^2^)	11.37	7.97	0.154	−4.65	10.77	0.666
Physical activity (0: sedentary, 1: active)	−20.06	13.10	0.127	−10.98	11.78	0.352
Smoking (0: non-smoke, 1: smoker)	−39.93	10.65	<0.001	−2.06	11.43	0.857
Maternal alcohol consumption (0: no, 1: yes)	−18.39	13.34	0.169	−5.11	16.08	0.751
Fish and seafood consumption (g/day)	0.34	0.13	0.011	0.223	0.172	0.195
Intervention Group (iron supplementation dosage)						
40 mg/day	Ref	-	-	Ref	-	-
20 mg/day	−3.57	10.65	0.738	−11.75	11.37	0.302
80 mg/day	22.46	8.66	0.010	−7.05	10.51	0.503
	R^2^_CX100_ = 10.20	F_18,334_ = 3.22	*p* < 0.001	R^2^_CX100_ = 3.6	F_18,236_ = 1.52	*p* = 0.084
Total n-3 PUFA						
Constant	240.59	28.99	<0.001	256.72	34.42	<0.001
Educational level						
Low	Ref	-	-	Ref	-	-
Medium	17.10	12.24	0.163	1.78	13.13	0.892
High	30.08	12.96	0.021	16.47	14.31	0.251
Age group						
<25 years	Ref	-	-	Ref	-	-
25–30 years	24.10	16.19	0.132	39.82	16.31	0.015
>30 years	41.49	15.58	0.008	51.88	15.71	0.001
BMI (0: < 25 kg/m^2^, 1: ≥ 25 kg/m^2^)	10.66	10.28	0.301	−10.43	12.83	0.417
Physical activity (0: sedentary, 1: active)	−22.93	16.69	0.170	−18.41	12.53	0.143
Smoking (0: non-smoke, 1: smoker)	−48.75	13.75	<0.001	0.763	13.63	0.955
Maternal alcohol consumption (0: no, 1: yes)	−20.63	17.24	0.232	−4.01	19.15	0.834
Fish and seafood consumption (g/day)	0.489	0.175	0.006	0.35	0.20	0.086
Intervention Group (iron supplementation dosage)						
40 mg/day	Ref	-	-	Ref	−	−
20 mg/day	−3.17	13.75	0.817	−18.91	13.50	0.163
80 mg/day	30.37	11.21	0.007	−11.35	12.44	0.363
	R^2^_CX100_ = 11.00	F_18,333_ = 3.41	*p* < 0.001	R^2^_CX100_ = 5.0	F_18,236_ = 1.74	*p* = 0.033

B, unstandardised coefficient; SE, standard error. Ref, reference category. Level of statistical significance *p* > 0.05. Variables included in multiple linear regression: educational level, group of age, BMI, smoking status, maternal alcohol consumption, physical activity, diet quality, consumption of fish and seafood, nuts, red and processed meat, lean meat, eggs, bakery and oil (including variables in the first or third trimester according to the timing of fatty acids assessment) and intervention group (iron supplementation of 40 mg/day, 20 mg/day or 80 mg/day). The variables not shown in the table were not significant.

**Table 4:** Multiple linear regression of potential factors related to maternal serum n-6/n-3 and AA/EPA ratios.

	Fatty Acids Serum Profile First Trimester			Fatty Acids Serum Profile Third Trimester		
	B	SE	*p*	B	SE	*p*
n-6/n-3 ratio						
Constant	17.13	1.50	<0.001	33.93	4.95	<0.001
Educational level						
Low	Ref	-	-	Ref	-	-
Medium	−1.19	0.64	0.063	−1.43	1.87	0.444
High	−3.30	0.67	<0.001	−5.26	2.03	0.010
Age group						
<25 years	Ref	-	-	Ref	-	-
25–30 years	0.68	0.85	0.421	0.06	2.32	0.977
>30 years	−0.17	0.82	0.828	−2.57	2.24	0.251
BMI (0: < 25 kg/m^2^, 1: ≥ 25 kg/m^2^)	1.15	0.53	0.033	1.98	1.83	0.282
Physical activity (0: sedentary, 1: active)	1.54	0.87	0.078	1.28	1.78	0.471
Smoking (0: non-smoke, 1: smoker)	2.70	0.72	<0.001	4.58	1.93	0.019
Maternal alcohol consumption (0: no, 1: yes)	0.57	0.90	0.526	−1.54	2.78	0.579
Fish and seafood consumption (g/day)	−0.03	0.01	<0.001	−0.07	0.02	0.008
Intervention group (iron supplementation dosage)	−0.02	0.68	0.769			
40 mg/day	Ref	-	-	Ref	-	-
20 mg/day	0.68	0.72	0.344	−1.26	1.90	0.507
80 mg/day	−1.06	0.58	0.70	−0.04	1.76	0.979
	R^2^_CX100_ = 16.6	F _18,334_ = 4.89	*p* < 0.001	R^2^_CX100_ = 7.70	F_18,237_ = 2.18	*p* = 0.004
AA/EPA ratio						
Constant	37.45	5.59	<0.001	63.08	12.31	<0.001
Educational level						
Low	Ref	-	-	Ref	-	-
Medium	−5.46	2.38	0.023	−9.24	4.69	0.050
High	−7.42	2.51	0.003	−19.21	5.07	<0.001
Age group						
<25 years	Ref	-	-	Ref	-	-
25–30 years	−6.39	3.16	0.044	−11.05	5.84	0.060
>30 years	−10.34	3.01	0.001	−12.70	5.61	0.024
BMI (0: < 25 kg/m^2^, 1: ≥ 25 kg/m^2^)	4.08	1.99	0.042	0.318	4.56	0.945
Physical activity (0: sedentary, 1: active)	5.73	3.20	0.074	0.31	4.56	0.945
Smoking (0: non-smoke, 1: smoker)	3.82	2.64	0.148	−2.16	4.85	0.656
Maternal alcohol consumption (0: no, 1: yes)	0.54	3.35	0.871	0.25	6.87	0.970
Fish and seafood consumption (g/day)	−0.16	0.03	<0.001	−0.26	0.70	<0.001
Bakery consumption (g/day)	0.05	0.03	0.132	0.19	0.073	0.008
Intervention Group (iron supplementation dosage)						
40 mg/day	Ref	-	-	Ref	-	-
20 mg/day	−0.13	2.65	0.959	−1.07	4.86	0.825
80 mg/day	−4.35	2.16	0.045	−0.198	4.38	0.964
	R^2^_CX100_ = 16.9	F _18,329_ = 7.23	*p* < 0.001	R^2^_CX100_ = 13.5	F_18,233_ = 3.17	*p* < 0.001

B, unstandardised coefficient; SE, standard error. Ref, reference category. Level of statistical significance *p*> 0.05. Variables included in multiple linear regression: educational level, group of age, BMI, smoking status, maternal alcohol consumption, physical activity, diet quality, consumption of fish and seafood, nuts, red and processed meat, lean meat, eggs, bakery and oil (including variables in the first or third trimester according to the timing of fatty acids assessment) and intervention group (iron supplementation of 40 mg/day, 20 mg/day or 80 mg/day). The variables not shown in the table were not significant.
